# Postmenarche growth: cohort study among indigenous and non-indigenous Chilean adolescents

**DOI:** 10.1186/s12889-015-1389-y

**Published:** 2015-01-31

**Authors:** Hugo Amigo, Macarena Lara, Patricia Bustos, Sergio Muñoz

**Affiliations:** Department of Nutrition, Faculty of Medicine, Universidad de Chile, Independencia 1027, Santiago, Chile; Department of Public Health, Universidad de la Frontera, Temuco, Chile

**Keywords:** Cohort study, Menarche, Growth, Indigenous adolescents

## Abstract

**Background:**

In Chile, indigenous and non-indigenous schoolchildren have the same stature when they begin school but indigenous adults are shorter, indicating the importance of analyzing growth during puberty. The aim of this study was to compare the growth of indigenous and non-indigenous girls during the 36 months after menarche in Chile’s Araucanía Region.

**Methods:**

A concurrent cohort study was conducted to compare growth in the two ethnic groups, which were comprised of 114 indigenous and 126 non-indigenous girls who recently experienced menarche and were randomly selected. Height was measured at menarche and at 6, 12, 18, 24 and 36 months post-menarche. General linear models were used to analyze growth and a generalized estimating equation model was used to compare height at 36 months post-menarche.

**Results:**

At menarche, the Z-score of height/age was less for indigenous than non-indigenous girls (−0.01 vs. −0.61, p < 0.001). Indigenous girls grew at a slower rate than non-indigenous girls (6.5 vs. 7.2 cm, p = 0.02), and height at 36-months post-menarche reached −0.82 vs. -0.35 cm (p *<*0.001). In an adjusted model at 36 months post-menarche, indigenous girls were 1.6 cm shorter than non-indigenous girls (95% confidence interval: −3.13 to −0.04).

**Conclusions:**

The height of indigenous girls at menarche was lower than that of non-indigenous girls and they subsequently grew less, maintaining the gap between the two groups. At the end of the follow-up period, the indigenous girls were shorter than their non-indigenous peers.

## Background

Pubertal events represent a milestone in individuals’ growth and development. Previous studies have shown that in women, the onset of puberty begins with thelarche and a growth spurt starts soon after the onset of puberty, around the age of 12, depending of the population [1]. From that moment, the velocity of increment in height starts to decrease and stops completely three to four years after menarche [2].

Studies in Chile reveal differences in the stature of indigenous and non-indigenous women by the end of the growth period, with indigenous subjects being shorter than non-indigenous ones [3,4]. Nevertheless, there are findings that demonstrate that these differences are not present either at birth (cross-sectional study of the universe of newborns) or when children begin to attend elementary school (six years old), (cross-sectional study with a sample from two regions) [5,6]. Therefore, it is important to verify whether these differences appear during pubertal events or soon thereafter.

In Chile, 5% of the population is indigenous and of this group, 87% are Mapuche. The Mapuche (or Araucanos) mainly reside in rural areas of the Araucanía Region and in Chile’s capital, Santiago [7]. According to the Human Development Index, the socioeconomic conditions of this ethnic group are less favorable than those of non-indigenous Chileans, and this inequality persists in spite of the country’s economic progress in recent decades [8]. Inequality may have an effect on the indigenous population such that it is unable to express its full growth potential, resulting in members of this group reaching a shorter final stature than their non-indigenous peers [3].

This growth deficit may have negative consequences for child and adolescent development, causing difficulties in social insertion due to poorer test performance and an increased probability of living in poverty among this population [9] Thus, it may reflect the inability of less-developed societies to express the full genetic potential of their people. Because the indigenous population is at high social risk, interventions aimed at reducing biological and social inequalities should be a priority.

Given this background, the study of anthropometric characteristics of indigenous and non-indigenous subjects at menarche and their subsequent evolution is relevant. The objective of this study was to compare the growth of indigenous and non-indigenous girls in the Araucanía Region in Chile from menarche to 36 months post-menarche.

## Methods

### Study design

A concurrent cohort study was conducted to compare the growth over time in the two ethnic groups.

### Setting

Of the Araucanía Region’s 32 counties, six were excluded because the indigenous population was scarce. In each of the 26 remaining counties, schools were selected at random (n = 168) in order to identify girls who had experienced menarche no more than three months prior to the start of the study and to determine which ethnic group they belonged to. The “indigenous cohort” included only girls with three or four Mapuche surnames while the “non-indigenous cohort” included girls whose surnames (two on both the father’s side and mother’s side) were of Spanish origin. Adolescents with non-Spanish surnames or for whom one parent’s surname was not known were excluded from the study. A list of more than 7,000 Mapuche surnames that had been previously written and validated by Mapuche language experts [10] was used as a reference. Girls with a combination of surnames (Spanish and Mapuche), other surnames (neither Spanish nor Mapuche), and girls for whom at least one parent’s surname was not known were excluded from the study.

The study was based on multistage random sampling of participants by three geographical areas (coast, valley and mountains). In each county of each stratum a list of public schools was drawn, that included the total number of girls from 10 to 16 years old (n = 8504). This list was then screened to assess the girls’ eligibility to participate in the study (menarche and ethnicity). The source of information used was official registration records from the Ministry of Education.

The sample size was calculated considering a 0.05 level of significance, a test power of 80%, and using a one-sided test. The expected increase on average from menarche to 36 months post-menarche in indigenous girls was 6.7 vs. 7.4 cm for non-indigenous girls, (these data were obtained from previous studies in Chile and Mexico) [4,11] with effect size of 0.372 and a common standard deviation of 1.9 cm. The minimum number for each group was 90 females. The universe of girls who had menarche no more than three months prior to the start of the study was 240 (114 indigenous and 126 non-indigenous girls).

### Data collection

An initial data collection instrument was applied to all participants in school classrooms to collect the following data: surnames, date of birth and age at menarche (corroborated by their parents). Subsequently, girls who had menarche within the three months preceding the interview were selected to be anthropometrically measured every three months at their respective schools by healthcare professionals who were trained in accepted international protocols for anthropometric measurement [12]. An in-depth survey, with questions about family socioeconomic status, eating habits, physical activity and health conditions, was applied to the mothers or fathers of the girls who participated in the follow-up.

Stature was measured with a 1-mm precision Seca stadiometer. While they were being measured, the adolescents stood straight on a flat surface with their feet parallel to each other, in bare or stocking feet. Their heels and backs touched the wall. The subjects were instructed to look straight ahead. The head was positioned such that the lower edge of the bony orbit was in line with the groove at the top of the tragus of the ear. Weight was measured using electronic calibrated SECA scales to the nearest 100 grams with the subject wearing minimal clothing (T-shirt and trousers or blouse and skirt, and underwear), the feet together in the centre of the scales and looking forward.

### Analysis and data processing

A descriptive analysis of the variables was developed to characterize the cohorts, in which the parents’ schooling and the family socioeconomic level and age at menarche were determined. Socioeconomic level was defined using a matrix that included the head of household’s educational level (schooling categories) and household assets (gas-fueled water heater, computer, refrigerator, washing machine, microwave oven, and car), according to a socioeconomic classification that has been widely used and validated in Chile [13]. According to this matrix, the girls were classified in five socioeconomic levels: upper, middle-upper, middle, middle-lower and low. The sample did not contain any girl classified in the middle-upper and upper level.

The mean height was calculated at time of menarche and at 6, 12, 18, 24, 30 and 36 months post-menarche; the Z-scores for height/age and weight/age and BMI/age were calculated according to CDC/NCHS references (as recommended by the Chilean Health Ministry for evaluating the nutritional status of children 6 to 18 years of age) [14]. Variance analysis was used to evaluate differences between the ethnic groups.

Height increment from menarche up 36 months was determined by calculating the difference in stature at each interval with the stature of the previous interval. The same method was used to calculate the difference for the full period. It should be noted that measurements weren’t taken exactly at menarche but within 3 months of menarche, so a mathematical equation was used to calculate the exact point in time. The formula was used to calculate the growth rate every 6 months during the follow-up period.

To analyze growth, repeated measurement analysis was applied using the multivariate general linear model (GLM) [15]. This model was used to analyze a time effect on growth of both cohorts and determine whether this effect depends on ethnicity. In this model, the seven measurements taken (0, 6, 12, 18, 24, 30 and 36 months) were analyzed as a responsive or dependent variable (height and Z-score for height/age). Also, we decided to use the GEE model to analyze the ethnicity effect on height and Z-score for height/age from menarche to 36 months after because it allows us to adjust by potential confounders (SES and age at menarche).

The data from the models is available for each subject from both ethnic groups. Wilks’ lambda statistic was used to verify the statistical significance with respect to the null hypothesis (no variation over time) and in order to verify the parallelism of the growth curves a test was performed to determine whether the interaction between ethnicity and time was statistically significant. Finally, repeated measures analysis was performed using generalized estimating equation models (GEEM), which compared the height and z-score for height/age from menarche to 36 months post-menarche in indigenous adolescents to that of non-indigenous adolescents, adjusted and unadjusted for age at menarche and socioeconomic level. The independent variable was ethnicity and the dependent variable was the height or z-score for height/age.

The data was recorded using EPIDATA (double data entry) software and processed with SPSS version 20.0 and STATA version 10.0.

The project was approved by the Ethics Committee for Human Research of the Faculty of Medicine at the University of Chile. Prior to participating in the study, the girls and their mothers signed informed consent forms.

## Results

From the initial sample of 240 girls, 39 were not included in the follow-up because they either moved to other regions of the country, became pregnant or declined to participate. Of a theoretical 1680 measurements (7 times for each girl) there were 1523 measurements, reaching a participation rate of 90.1% among non-indigenous girls and 91.2% among indigenous girls.

The mean age at menarche was 4.3 months later in indigenous girls compared to non-indigenous girls (p = 0.003). Height at menarche was 2.2 cm higher in non-indigenous girls, so the Z-score of height at the time of this pubertal event was close to the median in non-indigenous girls but significantly lower among indigenous girls (Z-score 0.10 vs.-0.61). Although the mean weight at menarche was similar in the two groups (p = 0.073) the Z-score of BMI was higher in indigenous girls than non-indigenous girls (p = 0.017). The educational level of the parents of indigenous subjects was significantly lower than the educational level of the parents of non-indigenous girls (p = 0.001). For the full sample, the parents’ level of education was less than 10 years and the lowest average was found for the mothers of indigenous girls. Half of the indigenous girls were classified in the low socioeconomic level, while fewer than 10% of the non-indigenous girls were similarly classified. On the contrary, more than 46% of the non-indigenous schoolgirls belonged to the middle socioeconomic level, while only 8.5% of the indigenous adolescents belonged to this group (Table 1).

**Table 1:** **Anthropometric and general characteristics of indigenous and non-indigenous girls in the Araucanía Region, Chile**

**Characteristics**	**Non-indigenous (n = 126)**	**Indigenous (n = 114)**	***P value***
Age at menarche (months)			
$\overline{x}\pm \mathrm{S}\mathrm{D}$	146.7 ± 11.3	151.0 ± 10.8	0.003*
Height at menarche (cm)			
$\overline{x}\pm \mathrm{S}\mathrm{D}$	152.1 ± 5.7	149.9 ± 4.8	0.002*
Z-score for height/age at menarche			
$\overline{x}\pm \mathrm{S}\mathrm{D}$	−0.01 ± 1.02	−0.61 ± 0.81	< 0.001*
Weight at menarche (kg)			
$\overline{x}\pm \mathrm{S}\mathrm{D}$	49.6 ± 8.8	51.7 ± 9.2	0.073*
Z-score for BMI/age at menarche			
$\overline{x}\pm \mathrm{S}\mathrm{D}$	0.95 ± 1.1	1.27 ± 0.98	0.017*
Mother’s schooling (years)			
$\overline{x}\pm \mathrm{S}\mathrm{D}$	9.5 ± 2.8	6.6 ± 2.9	0.001 *
Father’s schooling (years)			
$\overline{x}\pm \mathrm{S}\mathrm{D}$	9.6 ± 2.8	7.2 ± 2.8	0.001*
Socioeconomic level			
%			
Middle	46.2	8.5	
Middle-low	44.4	41.5	< 0.001**
low	9.4	50.0	

$\overline{x}\pm \mathrm{S}\mathrm{D}$ = mean ± standard deviation.

*ANOVA test.

**χ ^2^ test.

**Figure 1:**
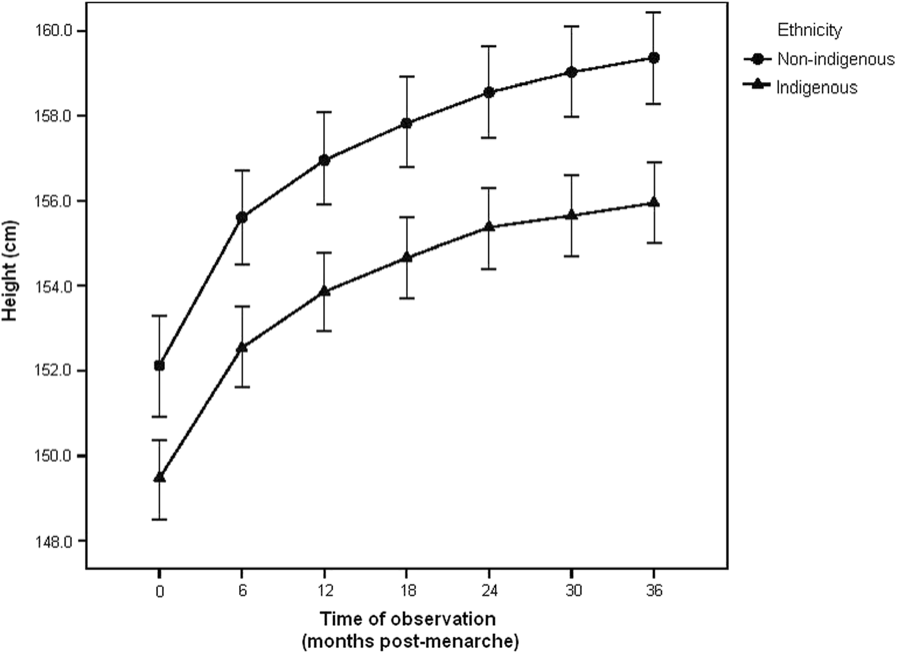
**Height according to time post-menarche in indigenous and non-indigenous girls.** At each time of observation, the differences in the means of the groups were statistically significant (p < 0.001). Wilks’ lambda for no time effect (p < 0.001). Wilks’ lambda for effect of time – ethnicity interaction (p < 0.023).

**Figure 2:**
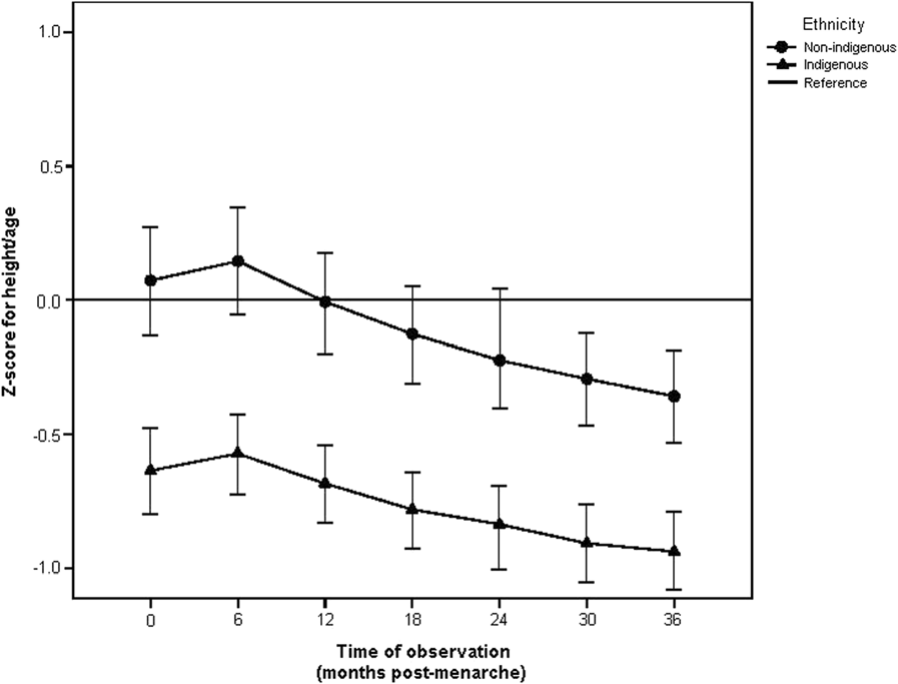
**Height/age (z- score) according to time post-menarche in indigenous and non-indigenous girls.** At each time of observation the differences in the means of the groups were statistically significant (p < 0.001). Wilks’ lambda for no time effect (p < 0.001). Wilks’ lambda for effect of time – ethnicity interaction (p < 0.112).

At the time of menarche, the indigenous adolescents were of significantly shorter stature: 2.2 cm shorter than non-indigenous girls (p < 0.001). This difference widened slightly as the months passed after menarche, reaching a difference of 3.2 cm at 36 months post-menarche (p < 0.001). The GLM for repeated measures indicated a significant increase in height over time in both groups (p < 0.001), although the non-indigenous girls presented a higher increase in height over time compared to their indigenous peers. At the different points of observation after menarche, height means were always higher for the non-indigenous adolescents than for the indigenous ones (Figure 1). When stature was standardized by age and sex (Z-score for height/age) the non-indigenous girls had higher values than the indigenous girls at each point of observation (p < 0.001) and the former group also had values close to the reference (p > 0.05), while indigenous girls always showed values below the reference (p < 0.001). Finally, at six months post-menarche, the Z-score for height began to decrease in both groups (p < 0.001) with slight parallelism in the curves (p = 0.112) (Figure 2).

Mean stature increased by 6.9 cm for the whole group from menarche until 36 months after this pubertal event, and was 0.7 cm higher for the non-indigenous group (p = 0.02). During the first six months post-menarche, there was a significantly smaller increase in height among the indigenous girls. After this period, the stature increment was similar for both ethnic groups, with the exception of the period from 24 to 30 months after menarche. Furthermore, a decrease in the growth rate in both cohorts was observed as the months passed after menarche (Table 2).

**Table 2:** **Height increment from 6 to 36 months post-menarche in indigenous and non-indigenous girls**

**Period**	**Growth (cm)**		P value*
	**Non-indigenous**	**Indigenous**	
0-6 months post-menarche	(n =117)	(n =108)	0.001
$\overline{x}\left[95\%\;\mathrm{C}\mathrm{I}\right]$	3.5 [3.3 to 3.7]	3.0 [2.8 to 3.2]	
6-12 months post-menarche	(n =117)	(n =105)	0.856
$\overline{x}\left[95\%\;\mathrm{C}\mathrm{I}\right]$	1.3 [1.1 to 1.5]	1.3 [1.2 to 1.5]	
12-18 months post-menarche	(n =113)	(n =104)	0.546
$\overline{x}\left[95\%\;\mathrm{C}\mathrm{I}\right]$	0.8 [0.7 to 1.0]	0.8 [0.6 to 0.9]	
18-24 months post-menarche	(n =109)	(n =101)	0.719
$\overline{x}\left[95\%\;\mathrm{C}\mathrm{I}\right]$	0.7 [0.6 to 0.9]	0.7 [0.6 to 0.8]	
24-30 months post-menarche	(n =108)	(n =100)	0.010
$\overline{x}\left[95\%\;\mathrm{C}\mathrm{I}\right]$	0.5 [0.4 to 0.6]	0.3 [0.2 to 0.4]	
30-36 months post-menarche	(n =105)	(n =96)	0.674
$\overline{x}\left[95\%\;\mathrm{C}\mathrm{I}\right]$	0.3 [0.2 to 0.4]	0.3 [0.2 to 0.4]	
Total:			
0-36 months post-menarche	(n =105)	(n =96)	0.020
$\overline{x}\left[95\%\;\mathrm{C}\mathrm{I}\right]$	7.2 [6.8 to 7.7]	6.5 [6.0 to 6.9]	

$\overline{x}\left[95\%\;\mathrm{C}\mathrm{I}\right]$ = Mean 95% Confidence Interval.

*ANOVA test.

An analysis of the height and Z-score for height/age from menarche to 36 months post-menarche using the GEEM showed that indigenous girls were significantly shorter than non-indigenous girls both in the unadjusted model and in the model adjusted for socioeconomic level, age at menarche and anthropometric value at menarche. In the latter model, the difference was lower but nevertheless significant (Table 3).

**Table 3:** **Effect of ethnicity on height, Z score for height/age from menarche to 36 months post-menarche among adolescents of the Araucanía Region**

	**Without adjustment**		Adjusted**	
	**Indigenous**	***P value***	**Indigenous**	***P value***
Height (cm) β coefficient [95% CI]	−2.7 [−4.02 to −1.39]	< 0.001	−1.9 [−3.32 to −0.51]	0.008
Z score for height/age β coefficient [95% CI]	−0.6 [−0.79 to −0.36]	< 0.001	−0.3 [−0.50 to −0.07]	0.008

Repeated measurements model (GEE).

**Adjusted by age at menarche and socioeconomic level (using the middle level as reference).

## Discussion

In this study tracking two cohorts, the indigenous girls were shorter than non-indigenous ones at menarche and the height increase during the 36 months after menarche was also lower for the indigenous girls. The study found a decrease in the rate of growth after menarche, and a tendency for growth to stop two years post-menarche in both ethnic groups. Finally, the indigenous and non-indigenous girls showed a smaller increase in height than the international reference, suggesting that by the end of adolescence there will be a deficit in height.

Interestingly, the indigenous girls in the study were of shorter stature than the non-indigenous girls at the time of menarche, even though previous studies had found no stature differences at birth and upon entering school [5,16]. Thus, it may be deduced that this difference begins during the period between the onset of puberty and menarche. These elements indicate the need to specify the moment at which stature differences between ethnic groups start to develop. It is also possible that indigenous girls do not reach the stature of non-indigenous girls because of exposure to less favorable socioeconomic conditions, which inhibit their ability to fully express their growth potential [17,18]. These stature differences at the time of menarche may reflect not only environmental conditions to which the studied cohorts have been exposed, but also the effects of other factors, since some studies have found that the age at which pubertal development begins and ends, as well as the amount of growth that occurs during this period, are influenced by genetic factors [19,20]. Although it may be the case that there are differences in the simple designs of the various studies of the indigenous population at different ages in Chile, we don’t believe that this is a decisive explanatory factor.

After 36 months of follow-up, the stature of indigenous adolescents was shorter than that of non-indigenous girls, which was expected given that the indigenous adolescents were shorter at the time of menarche. Furthermore, the indigenous adolescents showed less growth than non-indigenous adolescents during the three-year period in which they were monitored, especially in the first six months post-menarche. This makes sense if one takes into consideration that the Mapuche girls in the study were older than the non-Mapuche girls and thus were at a stage in which their growth was decelerating more rapidly. This translated, therefore, into a lower increase in stature.

The mean stature of the adolescents at 36 months post-menarche was shorter than the international reference [14]. This reference is based on a study of girls in the United States that used a cross-sectional design. Another notable finding from the data that deserves more careful study is that at two years post-menarche, growth in height tends to stop among both indigenous and non-indigenous girls. This growth deceleration is demonstrated in this study and may support the hypothesis that the final stature of the adolescents included in the study will not vary significantly from their height at 36 months post-menarche. Nevertheless, the international reference indicates that after this period growth does continue, suggesting that the growth of these girls will ends at a shorter stature than the international reference [4]. It should be noted that in this population there were no girls from the middle-upper or upper socioeconomic levels and that the simple was taken from public schools, so its external validity is limited to this population. These finding merits further investigation because Chile’s status as a country with an intermediate level of development may be reflected by adult height. Therefore, in the future it may be possible to observe height increments correlated to improvement in socioeconomic conditions, as seen in industrialized countries [21-23].

The stature reported in this study is higher than the previous height published for indigenous and non-indigenous adults in the country [4,24], reaffirming that the latest generations are taller than previous ones, a phenomenon that has also been observed in other countries [25-27]. Furthermore, it has been observed that Chilean Mapuche adolescents are taller than their indigenous peers in Latin America [28-30]. The taller stature of Chile’s indigenous people may be due to improvements in social conditions in the country, particularly a decrease in poverty rates, which may have had a positive impact on the indigenous population [31].

Classification in a specific socioeconomic level was used as a control variable because of the association between low socioeconomic level and low stature, which had been observed in previous studies in Chile [16,31-34]. Due to the high proportion of poverty, the indigenous girls would be expected to be shorter at the onset of puberty and to present later onset of puberty and subsequently later age at menarche. To consider these aspects, it was necessary to adjust by these variables to control for the confounder or modification effect. This study verified that when these variables were included in the multivariate model, the effect of ethnic group remained and this confirms that stature differences are produced at this age, which merits further study. It is important to note that although the socioeconomic index used has been validated for the Chilean population, it has not been validated specifically for the indigenous population, who may have particularities due to their customs and lifestyle. Currently, indigenous adolescents do not manifest living conditions that are dissimilar to non-indigenous adolescents, since there has been a progressive loss of ethnic traditions and customs [35].

One of the limitations of this study is that the subjects have not yet reached their final stature although further growth is expected to be minimal. However, it is worth mentioning that this is one of the first studies to track the growth of indigenous and non-indigenous adolescents. Another limitation of the study is that the subjects’ growth from the start of pubertal development (thelarche) was unknown, but this aspect is interesting since this phase could produce different growth. In our study, we verified that a stature difference between the ethnic groups already existed at menarche.

Moreover, one of the study’s strengths is the use of surnames as an indicator of indigenous origin, a method developed by linguists who specialize in the Mapuche language [9]. In the country, a useful way to identify indigenous ethnicity is by surname, as Mapuche surnames are quite unique and based on elements of nature. In Chile it is difficult to use other markers such as language or geographic location because these have been lost over time.

It should also be mentioned that the results of this study are externally valid because the schools and girls from the Araucanía Region who participated were selected randomly. The size of the sample was adequate for obtaining valid conclusions from the post-menarche anthropometric changes in both ethnic groups. At the same time, menarche had occurred recently for the study participants and was corroborated by the adolescents’ mothers; this short time lapse reduces the potential for memory bias. It should also be mentioned that this study was conducted by qualified professionals who were trained to use standard methods and were constantly supervised while obtaining the anthropometric measurements.

This study is one of a series of studies on indigenous and non-indigenous females that include size at birth [5], growth during infancy [36], stature in primary school [6] and parental height [3,4]. This study of adolescent Mapuche and non-Mapuche girls complements this line of research.

From the standpoint of policies and programs, this study underscores the importance of monitoring growth in height during different stages of life, including adolescence.

## Conclusion

The indigenous adolescents were shorter than non-indigenous ones at menarche; the height increase during the 36 months after menarche was also smaller for the indigenous girls. There was a decrease in the rate of growth after menarche and a tendency for growth to completely stop two years post-menarche in both ethnic groups. Finally, girls from both groups showed a lower increase in height than the international reference, suggesting that by the end of adolescence there will be a deficit in height.
